# Plant immune receptor pathways as a united front against pathogens

**DOI:** 10.1371/journal.ppat.1011106

**Published:** 2023-02-09

**Authors:** Minhang Yuan, Boying Cai, Xiu-Fang Xin

**Affiliations:** 1 National key Laboratory of Plant Molecular Genetics, CAS Center for Excellence in Molecular Plant Sciences, Institute of Plant Physiology and Ecology, Chinese Academy of Sciences, Shanghai, China; 2 University of the Chinese Academy of Sciences, Beijing, China; 3 CAS-JIC Center of Excellence for Plant and Microbial Sciences (CEPAMS), Institute of Plant Physiology and Ecology, Chinese Academy of Sciences, Shanghai, China; Shanghai Center for Plant Stress Biology, CHINA

Plants evolved the innate immune system to activate disease resistance mechanisms and fend off microbial invaders. This system comprises two major signaling cascades initiated by two classes of immune receptors, the cell-surface immune receptors, also named pattern recognition receptors (PRRs), and intracellular immune receptors, also named nucleotide-binding domain leucine-rich repeat receptors (NLRs). PRRs and NLRs possess distinct biochemical activities and are activated via largely independent mechanisms. And yet, the downstream immune responses and outputs are strikingly similar, suggesting connectivity and convergence between the two pathways. Indeed, recent studies significantly advanced our understanding of the intimate relationship of interdependence and mutual potentiation between the two cascades. A united view of plant innate immunity is emerging.

## Cell-surface and intracellular receptors: Two routes of pathogen recognition

PRRs typically recognize the conserved pathogen/microbe/damage-associated molecular patterns (PAMPs/MAMPs/DAMPs), such as bacterial flagellin, fungal cell wall-derived chitin, and plant elicitor peptides (PEPs), leading to pattern-triggered immunity (PTI) [1]. Based on the type of C-terminal domain, PRRs are divided into receptor-like kinases (RLKs) and receptor-like proteins (RLPs), which, upon ligand binding, both recruit the somatic embryogenesis receptor kinase (SERKs) coreceptors to form a receptor complex and further activate, via *trans*-phosphorylation, the intracellular receptor-like cytoplasmic kinases (RLCKs); however, the signaling downstream RLKs and RLPs are partially different [1]. RLCKs serve as a central signaling hub and activate a range of substrate proteins to trigger downstream responses, including calcium (Ca^2+^) influx, reactive oxygen species (ROS) burst, mitogen-activated protein kinase (MAPK) cascade, transcriptional reprogramming, and stomatal closure [2]. On the other hand, NLRs usually detect, directly or indirectly, effectors, which are often virulence proteins produced by pathogens to promote infection, and lead to a stronger amplitude of immune responses collectively called effector-triggered immunity (ETI) [3,4]. NLRs can be divided into coiled-coil-type NLRs (CNL), Toll/interleukin-1 receptor/Resistance protein (TIR)-type NLRs (TNLs) and the Resistance to powdery mildew 8-like domain (RPW8)-type NLR (RNLs), based on the N-terminal domain. Based on functions, NLRs can be divided into “sensor NLRs” that recognize effectors and “helper NLRs” that act downstream of sensor NLRs in the ETI signaling [5]. At least two types of helper NLRs, RNL and NLR Required for Cell death (NRC), have been identified in plants. RNLs function downstream of TNLs and some CNLs and are conserved in diverse plant species, whereas the NRCs represent a specific clade of CNLs in Solanaceae plants and are required for sensor CNLs- and PRRs-mediated signaling [6]. Latest breakthrough studies show that CNL- and RNL-type receptor complexes, such as ZAR1 and NRG1 “resistosomes”, assemble into calcium-permeable channels upon effector recognition [7–9]. Differently, TNL resistosomes display an NADase activity and produce two groups of signaling molecules, 2′-(5″-phosphoribosyl)-5′-adenosine diphosphate/monophosphate (pRib-ADP/AMP) and ADP-ribosylated adenosine triphosphate/ADPr-ADPR (ADPr-ATP/di-ADPR), which mediate the formation of two distinct EDS1 dimer-helper NLR complexes (i.e., EDS1-PAD4-ADR1 and EDS1-SAG101-NRG1) to promote immune signaling destined for pathogen restriction and cell death, respectively [10–15]. In addition, the TIR domain of TNLs also acts as 2′,3′-cAMP/cGMP synthetases by hydrolyzing RNA/DNA, and the cNMP products play an important role in TIR signaling [16]. NLR activation or ETI usually confers strong resistance in plants against pathogens and, in many cases, culminates in fast cell death (hypersensitive response (HR)).

## An emerging theme of cooperation between cell-surface and intracellular immune receptor pathways

**Fig 1:**
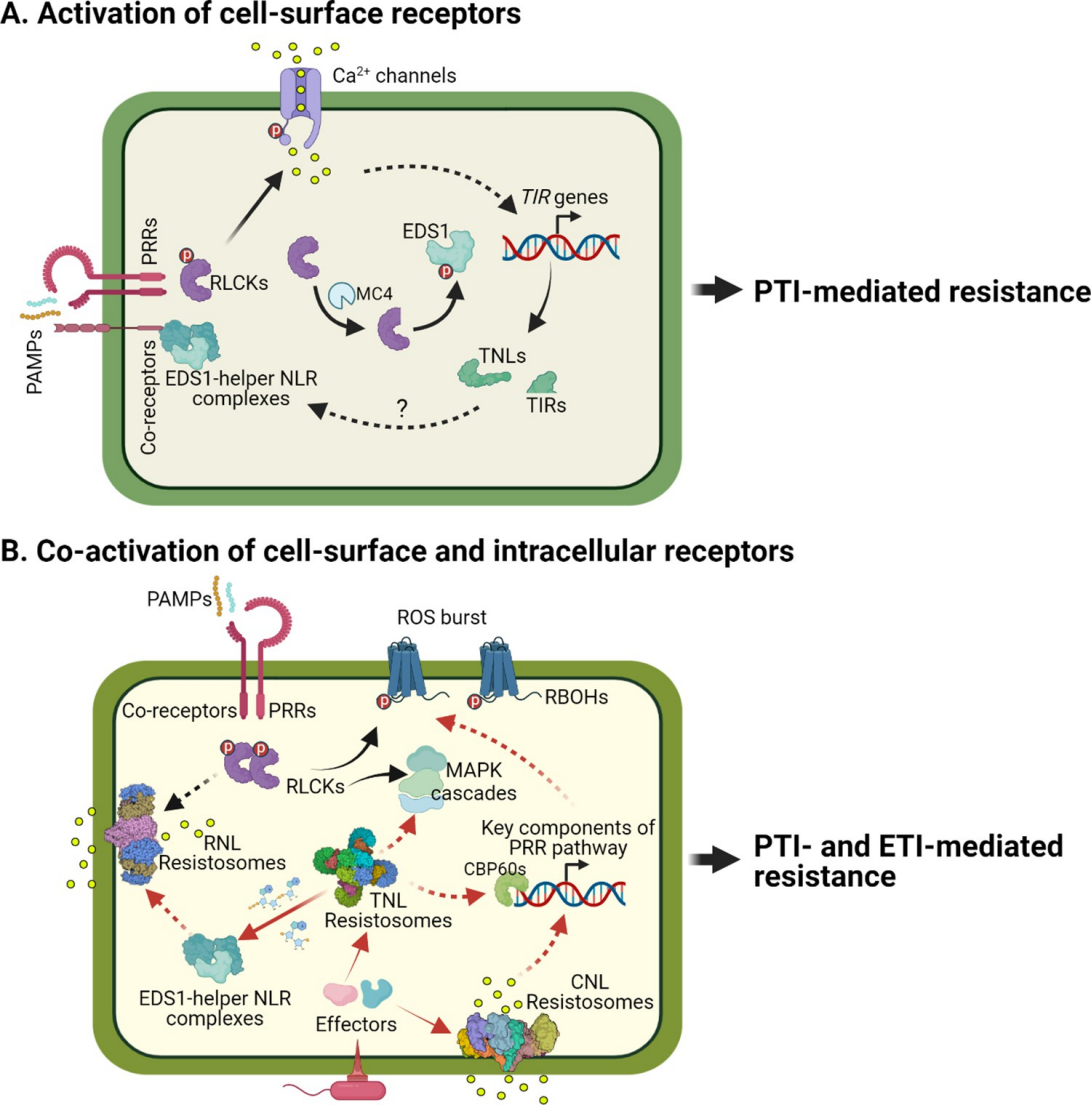
Schematic models for synergies between cell-surface and intracellular receptor-mediated signaling. **(A)** Upon ligand perception, PRRs recruit coreceptors (e.g., BAK1, CERK1) to phosphorylate each other and the downstream RLCKs. The activated RLCKs further induce Ca^2+^ influx by phosphorylating Ca^2+^ channels. Studies show that PTI up-regulates the transcription of TNL or TIR domain-containing genes, which requires PRR-initiated calcium flux. The cell-surface localized coreceptor SOBIR1 interacts with EDS1-helper NLR complexes to form supramolecular complexes in a ligand-independent manner, to potentially regulate PTI. Some RLCK proteins (e.g., PBL19) are cleaved by metacaspases like MC4 to generate truncated RLCKs, which phosphorylate EDS1 in the cytoplasm to boost plant resistance. **(B)** Activated TNL resistosomes serve as NAD^+^-cleaving enzymes and 2′,3′-cAMP/cGMP synthetase. NAD^+^-derived small molecules are produced and activate EDS1-helper NLR complexes. CNL and helper NLR (or RNL) resistosomes form calcium-permeable channels triggering immune signaling. A recent study suggests that the formation of RNL resistosome requires both PRR and TNL signaling. Similarly, activation of RBOHD-mediating ROS burst relies on both PRR and NLR signaling. Activation of MAPK cascades by TNLs requires PRR signaling. ETI increases the transcript and protein levels of many key components of PRR pathway, possibly through members of CBP60 transcription factor family. Black arrows indicate the regulation by cell-surface receptor pathway, and red arrows indicate the regulation by intracellular receptor pathway. Solid arrows indicate direct effects, and dashed ones indicate indirect effects where the mechanism is unclear. Created with BioRender.com. CNL, coiled-coil-type NLR; ETI, effector-triggered immunity; MAPK, mitogen-activated protein kinase; NLR, nucleotide-binding domain leucine-rich repeat receptor; PAMP, pathogen-associated molecular pattern; PPR, pattern recognition receptor; PTI, pattern-triggered immunity; RLCK, receptor-like cytoplasmic kinase; RNL, Resistance to powdery mildew 8-like domain (RPW8)-type NLR; ROS, reactive oxygen species; TIR, Toll/interleukin-1 receptor/Resistance protein; TNL, TIR-type NLR.

Because of the very different activation mechanisms, PRR and NLR signaling had been mostly studied as separate pathways. Early hints of their crosstalk came from the observations of shared physiological outputs; for example, activation of PTI alone or “PTI+ETI” can both lead to MAPK phosphorylation, ROS burst, Ca^2+^ influx, transcriptional reprogramming, and production of defense hormones, although ETI responses are more often associated with a higher amplitude and more sustained nature [17,18]. In addition, deficiency in the PRR signaling pathway, e.g., knocking out of the flagellin-encoding gene *fliC* in *P*. *syringae* pv. *Tabaci* 6505 or mutation of the coreceptor proteins BAK1 and BKK1 in *Arabidposis*, significantly reduces disease resistance mediated by certain NLRs (e.g., RPP2 and RPP4) [19,20], suggesting the importance of PRR pathway for ETI. Recently, two studies dissected the role of PRR and NLR signaling in stimulating ETI-associated immune responses, by uncoupling the two pathways using PRR mutant plants and transgenic plants expressing ETI-eliciting effectors, and uncovered a mutual potentiation mechanism between the two cascades [21,22]. Both studies show that PRR/coreceptors are indispensable for ETI-associated signaling events (e.g., ROS and/or MAPK phosphorylation), disease resistance, and HR [21,22]. Intriguingly, RBOHD, a NADPH oxidase that mediates ROS production, is coregulated by the two pathways, in that NLR signaling induces its transcript/protein level and PRR signaling is required for a full-scale RBOHD phosphorylation important for its activity (Fig 1) [21]. In addition, ETI-associated MAPK phosphorylation triggered by activation of RPS4/RRS1, a TNL complex, is completely dependent on PRR signaling, indicating that MAPK cascade in TNL signaling is also coregulated by two pathways [22]. Notably, the CNL RPS2-mediated MAPK phosphorylation seems to be largely independent of PRRs [21,22], suggesting divergence in coordination mechanisms of CNLs and TNLs. Furthermore, NLR signaling rapidly up-regulates the transcript and protein levels of many key components of PRR pathway, including BAK1 and RLCKs (Fig 1) [21,22], suggesting a “reboosting” of PTI by NLR signaling and a mutual amplification between the two pathways.

## The “EDS1-helper NLR complex” as a key intersecting point of PTI and ETI

As mentioned above, the EDS1-PAD4 and EDS1-SAG101 dimers interact with helper NLRs to transduce the signal from TNL receptors. Interestingly, recent studies suggest the “EDS1-helper NLR complex” as a key converging point of PTI and ETI. Two studies demonstrate that the EDS1, PAD4, and helper NLRs are important for cell-surface receptor (i.e., the RLK-type flagellin receptor FLS2 and the RLP-type oomycete nlp20 receptor RLP23)-mediated signaling and resistance [23,24]. Interestingly, these studies show that the EDS1-helper NLR complex is essential for RLP23-mediated and, to a lesser extent, FLS2-mediated PTI resistance against pathogens. Both RLK- and RLP-induced *FMO1*/*SARD1* expression and SA production require the EDS1-helper NLR complex; however, the EDS1-helper NLR complex only contributed to RLP- but not RLK-mediated ethylene production, ROS burst, and callose deposition [23,24]. In addition, Tian and colleagues report that treatment of plants with PAMPs rapidly induces the expression of many TIR genes, including TNLs and genes containing TIR domain (Fig 1) [24]. This up-regulation of NLRs in PTI was supported by other studies as well [25,26]. Pruitt and colleagues show that the cell-surface coreceptor SOBIR1 interacts with EDS1-PAD4-ADR1 in a ligand-independent manner, suggesting the formation of supramolecular complexes at the plasma membrane (Fig 1) [23]. Related to this, a previous study shows that the cell-surface receptor FLS2 physically associates with different NLR receptors, including RPS2, RPS5, and RPM1, in co-IP experiments [27], although how this affects PTI/ETI signaling is still unclear. More recently, Feehan and colleagues show that activation of TNLs triggers EDS1-SAG101-NRG1 interaction, and, importantly, PRR signaling is required for the formation of a putative oligomeric “EDS1-SAG101-NRG1” resistosome important for ETI [28], providing another potential mechanism of ETI’s dependence on PTI.

In addition, another study found that the RLCK PBL19, after metacaspase processing, specifically interacts with and phosphorylates EDS1 protein after treatment of fungal chitin in *Arabidopsis* [29]. The phosphorylated EDS1 protein accumulates in the cytoplasm and contributes to plant antifungal immunity (Fig 1) [29]. Whether the phosphorylated EDS1-mediated signaling is dependent on PAD4 or SAG101 remains to be determined. Notably, PBL19 also regulates plant immunity initiated by a variety of cell-surface receptors, including FLS2/BAK1 and RLP23/SOBIR1 [24,30]. Thus, this study reveals another regulatory layer by which EDS1 intercepts the PRR and NLR signaling cascades.

In addition to helper NLRs, very recent studies found that CSA1, a TNL protein, and its partner TNL CHS3 are in a complex with the PRR coreceptor BAK1 and its negative regulator BIR3 [31,32]. Furthermore, CSA1/CHS3 guard the integrity of BAK1 complex and trigger ETI-type cell death in the absence of BAK1/BIR3 complex [31,32]. Interestingly, CSA1 also contributes to plant basal immunity (e.g., against non-ETI-eliciting pathogens) [31], suggesting that CSA1 functions as a convergence point of PTI and ETI.

## Transcription regulators at the interface of PTI and ETI

In addition to the upstream signaling components involved in “PTI-ETI interplay” (as described above), components mediating downstream responses, such as RBOHD, MAPK cascade, and defense hormone pathways, also play dual roles in PTI and ETI resistance [21,22,33]. In addition, transcriptome analysis show that activation of PRRs (e.g., FLS2 and EFR) and NLRs (e.g., RPS2 and RPM1) induces highly similar gene sets [17], and the expression levels of some genes are synergistically regulated by PRRs and NLRs [21,22]. These suggest that common transcription elements regulate both signals. SARD1 and CBP60g, two members of CBP60 transcription factor family, are induced during PTI and ETI and important for plant resistance [21]. Consistent with this, chromatin-immunoprecipitation sequencing data revealed that many key components involved in PRR and NLR signaling, including BAK1, BIK1, and CPK4, could be targeted by these two transcription factors [34], suggesting that SARD1/CBP60g are central transcriptional hubs in PRR and NLR pathways. Calmodulin-binding transcription activators (CAMTAs) are proposed to function as transcriptional repressors of *SARD1/CBP60g* at the resting stage, and this repressor activity is released to allow the downstream gene expression during pathogen infection. The transcriptional reprogramming mediated by flg22 and NLR RPM1 was compromised in the *camta3* dominant mutant plants [35], and a recent study shows that CAMTA3 directly regulates the transcription of immune-related genes such as CBP60g, PEPR1, and RPS2 [36]. These results suggest that CAMTAs also regulate both PRR and NLR signaling. Together, these studies support a crucial role and converging point of the CAMTA-CBP60 module in regulating immune transcriptional programming.

## Conservation of PTI-ETI interactions in other species

**Fig 2:**
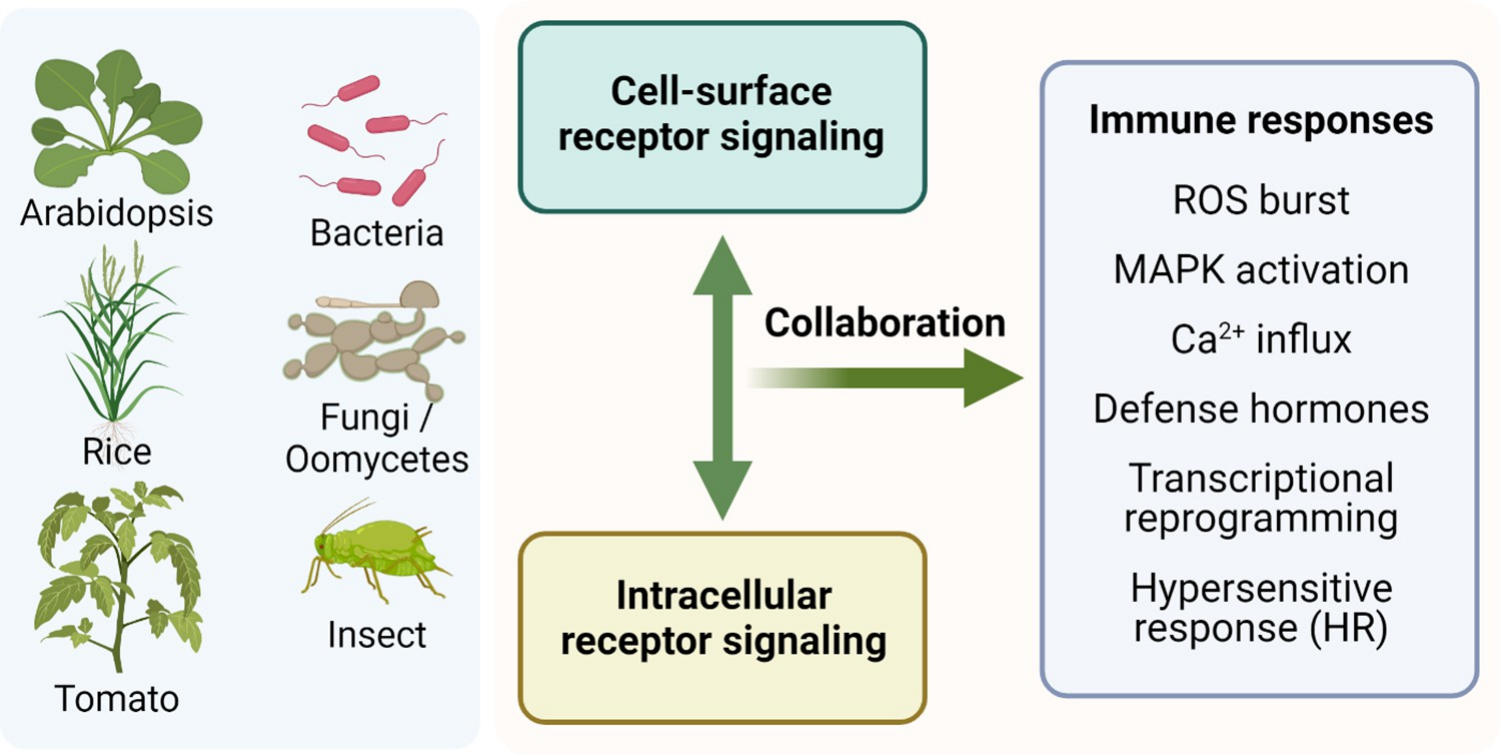
Conservation of synergies between cell-surface and intracellular receptor-mediated signaling in different plant pathosystems. Studies suggest a similar mode of synergistic interactions between cell surface and intracellular receptor pathways in some other plant species, but different forms of interplay between the two pathways may also exist. More studies are needed in the future to get a clear and full picture of plant immune networks in diverse species. Created with BioRender.com.

While the PTI-ETI interplay were mostly studied in the model plant *Arabidopsis thaliana* so far, an important question is whether the same principle applies to other plant species. Through an analysis of immune receptors in 350 plant species ranging from algal species to angiosperms, Ngou and colleagues show a strong positive linear correlation between the number of predicted cell-surface and that of intracellular receptors [37], suggesting a possible conserved synergy between cell-surface and intracellular immune receptors in diverse plant species. Indeed, several studies have revealed the interdependence between the two immune receptor pathways in other species. For example, the cell death induced by the expression of tomato RLP Cf-4, a cell-surface receptor, in *Nicotiana benthamiana* is dependent on the intracellular helper NLR protein NRC3 [38,39]. Similarly, in tomato, the intracellular components EDS1 and NDR1, which were long-known as key components of NLR signaling, are required for disease resistance conferred by the cell-surface receptor Ve1 [40]. Outside dicots, in the monocot rice, a recent study shows that a deubiquitinase PICI1, which is induced by fungal chitin and associates with NLR protein, promotes the biosynthesis of ethylene, by stabilizing methionine synthetase OsMETS1, to combat blast resistance [41]. The PICI1-OsMETS1-ethylene cascade is required for signaling by both cell-surface and intracellular receptors, suggesting a crucial junction between PRRs and NLRs in rice. In addition, the small GTPase OsRac1 forms two different immune receptor complexes with the PRR OsCERK1 and the NLR Pit to initiate rice immunity [42]. Thus, OsRac1 may be one of the key machineries that control both cell-surface and intracellular receptor signaling in rice. The above studies suggest a general principle of interplay between cell-surface and intracellular immune receptor pathways in different plant species (Fig 2).

On the other hand, the EDS1 complex does not seem to be required for certain cell surface receptor-mediated responses in *Nicotiana benthamiana*, since the EDS1 complex- or RNL-deficient lines show a normal response in Avr4- or flg22-triggered ROS burst and cell death [43]. Future studies are needed to fully decipher the potential conservation as well as different forms of PTI-ETI interplay across plant species.

## Future perspectives and remaining questions

While cell-surface and intracellular receptor-mediated signaling were initially identified as independent pathways leading to immunity, increasing evidence indicates the existence of multiple levels of sophisticated synergies between the two cascades, suggesting a revised model in which mutual potentiation of the two leads to a robust plant immunity. One emerging theme seems to be that some key immune components, such as RBOHD and “EDS1-helper NLR complex,” are coregulated by both PRR and NLR cascades for full activation. The “PTI-ETI synergy” provides an opportunity to reformulate the architecture of plant immune networks as well as add fresh perspectives in studying the functions of individual components. However, to date, the mechanisms by which the two layers of the immune system work together are very likely just the tip of the iceberg, and future investigations should help better understand the molecular mechanisms underlying the synergies. With key biochemical activities of NLR proteins and ETI signaling molecules uncovered recently, it will be interesting to determine whether PTI-ETI interplay is involved in regulating these activities/molecules. In addition, it’s important to understand how the PTI-ETI interplay works in other plant species, especially crops, which may suggest innovative strategies of breeding crops with durable and broad-spectrum disease resistance against infectious microbes.
